# GLP1 protects cardiomyocytes from palmitate-induced apoptosis via Akt/GSK3b/b-catenin pathway

**DOI:** 10.1530/JME-15-0155

**Published:** 2015-12

**Authors:** Ying Ying, Huazhang Zhu, Zhen Liang, Xiaosong Ma, Shiwei Li

**Affiliations:** 1 School of Medicine, Shenzhen University Diabetes Center, Shenzhen University, Shenzhen, 518060, China; 2 Department of Geriatric, Shenzhen Second People's Hospital, First Affiliated Hospital of Shenzhen University, Shenzhen, 518035, China

**Keywords:** palmitate, cardiomyocytes, apoptosis, b-catenin, GLP1, GLP1R, Akt, GSK3B

## Abstract

Activation of apoptosis in cardiomyocytes by saturated palmitic acids contributes to cardiac dysfunction in diabetic cardiomyopathy. Beta-catenin (b-catenin) is a transcriptional regulator of several genes involved in survival/anti-apoptosis. However, its role in palmitate-induced cardiomyocyte apoptosis remains unclear. Glucagon-like peptide 1 (GLP1) has been shown to exhibit potential cardioprotective properties. This study was designed to evaluate the role of b-catenin signalling in palmitate-induced cardiomyocyte apoptosis and the molecular mechanism underlying the protective effects of GLP1 on palmitate-stressed cardiomyocytes. Exposure of neonatal rat cardiomyocytes to palmitate increased the fatty acid transporter CD36-mediated intracellular lipid accumulation and cardiomyocyte apoptosis, decreased accumulation and nuclear translocation of active b-catenin, and reduced expression of b-catenin target protein survivin and BCL2. These detrimental effects of palmitate were significantly attenuated by GLP1 co-treatment. However, the anti-apoptotic effects of GLP1 were markedly abolished when b-catenin was silenced with a specific short hairpin RNA. Furthermore, analysis of the upstream molecules and mechanisms responsible for GLP1-associated cardiac protection revealed that GLP1 restored the decreased phosphorylation of protein kinase B (Akt) and glycogen synthase kinase-3b (GSK3b) in palmitate-stimulated cardiomyocytes. In contrast, inhibition of Akt with an Akt-specific inhibitor MK2206 or blockade of GLP1 receptor (GLP1R) with a competitive antagonist exendin-(9–39) significantly abrogated the GLP1-mediated activation of GSK3b/b-catenin signalling, leading to increased apoptosis in palmitate-stressed cardiomyocytes. Collectively, our results demonstrated for the first time that the attenuated b-catenin signalling may contribute to palmitate-induced cardiomyocyte apoptosis, while GLP1 can protect cardiomyocytes from palmitate-induced apoptosis through activation of GLP1R/Akt/GSK3b-mediated b-catenin signalling.

## Introduction

High levels of plasma free fatty acid seen in type 2 diabetes and obesity (Fraze *et al*. 1985) have been proposed as a potential mechanism for the development of diabetic/lipotoxic cardiomyopathy (Rodrigues *et al*. 1995, Zhou *et al*. 2000, Boudina & Abel 2007). Excessive saturated fatty acids such as palmitate induce cardiomyocyte apoptosis, which ultimately contributes to cardiac dysfunction and failure (Chiu *et al*. 2001, Narula *et al*. 2001). *In vitro* studies have indicated that the induction of apoptosis in cardiomyocytes by palmitate is associated with the mitochondria-dependent apoptotic pathway (Narula *et al*. 1999, Sparagna *et al*. 2000). However, molecular mechanisms underlying palmitate-induced cardiomyocyte apoptosis remain incompletely clarified.

Wnt and phosphatidylinositol-3-kinase (PI3K)/protein kinase B (Akt) signalling pathways converge through beta-catenin (b-catenin)-mediated regulation of cell survival/apoptosis in different cell types including cardiomyocytes (Ding *et al*. 2000, Fukumoto *et al*. 2001, Almeida *et al*. 2005). Nuclear b-catenin levels are regulated by glycogen synthase kinase-3b (GSK3b), which phosphorylates specific residues at serine 33, serine 37 and threonine 41 (Ser33/37/Thr41) of b-catenin for proteasomal degradation (Huelsken & Birchmeier 2001). In response to Wnt or PI3K/Akt signals, inactivation of GSK3B through phosphorylation of serine 9 residue (Ser9) (Ding *et al*. 2000) leads to the stabilization, cytosolic accumulation and subsequent translocation of b-catenin to the nucleus to transactivate the expression of specific genes involved in cell survival and proliferation (Behrens *et al*. 1996, Miller *et al*. 1999). Recent studies have suggested that the PI3K/GSK3B/b-catenin signalling pathway shows potential anti-apoptotic effects after myocardial infarction (MI) in rat ischemic preconditioning (Kaga *et al*. 2006) or post-conditioning (Wu *et al*. 2012) models. The importance of b-catenin in promoting cardiomyocyte survival in the process of preconditioning and healing after MI was further underscored by recent studies (Hahn *et al*. 2006, Thirunavukkarasu *et al*. 2008). Nonetheless, the role of b-catenin signalling in the context of palmitate-induced cardiomyocyte apoptosis has not been determined thus far.

Glucagon-like peptide 1 (GLP1) is an incretin hormone synthesized and secreted by intestinal L-cells and stimulates insulin secretion from pancreatic b-cells in a glucose-dependent manner (Baggio & Drucker 2007). Notably, GLP1 and its analogs have been demonstrated to exert cardioprotective actions in a few ischemia/reperfusion models, leading to improved cardiac function and enhanced cell survival (Ban *et al*. 2008, Noyan-Ashraf *et al*. 2009, Timmers *et al*. 2009). GLP1 analogs have also been reported to reverse the molecular pathology and cardiovascular dysfunction in obese mice (Noyan-Ashraf *et al*. 2013) and diabetic rats (Wang *et al*. 2013). Importantly, recent data have suggested that GLP1 is capable of exerting a direct anti-apoptotic effect against palmitate-induced apoptosis in murine HL-1 cardiomyocytes (Ravassa *et al*. 2011) and isolated primary mouse cardiomyocytes (Noyan-Ashraf *et al*. 2013). Hence, GLP1 is a potentially exciting strategy for cardioprotection in diabetes and obesity. Of interest, the ability of GLP1 in activating b-catenin signalling to enhance pancreatic b-cell proliferation has been recently described (Liu & Habener 2008). However, the role, if any, of these molecular mechanisms in the palmitate-mediated lipotoxic cardiomyopathy have not been examined.

Given that b-catenin signalling plays a vital role in promoting cardiomyocyte survival and that GLP1 exerts direct cardioprotective effects upon lipotoxicity, we hypothesized that GLP1 could protect cardiomyocytes from palmitate-induced apoptosis via the activation of b-catenin signalling. To test this hypothesis, the present study was designed to investigate whether b-catenin signalling provides a mechanistic link in palmitate-induced apoptosis of isolated neonatal rat cardiomyocytes. Furthermore, the molecular mechanism underlying the protective effects of GLP1 on cardiomyocytes upon palmitate stress was characterized.

## Materials and methods

### Animal, isolation and cultivation of neonatal rat cardiomyocytes

Neonatal Sprague–Dawley rats (1–3 days old) were purchased from the Experimental Animal Centre of Guangdong Academy of Medical Science (Guangzhou, China). Neonatal rat cardiomyocytes were isolated as previously described (Peng *et al*. 2003). The animal procedures were performed according to the Principles of Laboratory Animal Care and approved by the Shenzhen University Animal Care Committee (permit no. 201412043). Cardiomyocytes were maintained in DMEM containing 0.3 g/l glutamine, 4.5 g/l glucose and 10% heat inactivated fetal bovine serum (FBS).

### Experimental design

When neonatal rat cardiomyocytes reached ∼80% confluence, growth media were changed to serum-free media for 24 h. Cells were then incubated with palmitate (Sigma–Aldrich) pre-conjugated to BSA (Sigma–Aldrich) in 0.5% FBS–DMEM, in the absence or presence of GLP1 (Abcam, Cambridge, UK) alone, or in combination of GLP1 with exendin-(9–39) (Sigma–Aldrich), a GLP1 receptor (GLP1R) antagonist or MK2206 (Selleck, Trenton, NJ, USA), an Akt inhibitor, for up to 24 h. For drug interference, cardiomyocytes were pre-treated with the indicated reagents (GLP1, exendin-(9–39), MK2206) 1 h prior to palmitate treatment. The stock solution of 5 mM palmitate bound to 10% BSA was prepared using the methods described before (Cousin *et al*. 2001). The final concentration of palmitate in the stock solutions was determined before and after sterile filtration with a commercially available kit (Wako Chemicals, Neuss, Germany). For control incubations, 10% BSA was also prepared.

### Knock-down of b-catenin by adeno-short-hairpin RNA-b-catenin transduction

First, siRNA sequences against rat b-catenin (GenBank accession number: NM_053357) were designed using RNA structure 4.4 Software (Warrendale, PA, USA). Next, we generated the short-hairpin RNA (shRNA) against b-catenin through a DNA vector-based strategy. The pDC316-ZsGreen-sh-b-catenin vector was constructed by inserting annealed double-stranded oligo DNA with BamHI and EcoRI restriction enzyme sites (Table 1), into the pDC316-zsGreen-shRNA empty vector cut by the same enzyme. The knockdown efficiency of constructed plasmids was determined by transfection onto C2C12 cells using Lipofectamine 2000 (Invitrogen), followed by the examination of endogenous b-catenin mRNA expression with primers specific for *b-catenin* (Table 2) through quantitative real-time PCR. Last, the pDC316-ZsGreen-sh-b-catenin-1 vector showing the highest efficiency of b-catenin silencing was subsequently used to generate a recombinant adenovirus (Ad-sh-b-catenin) by using pBHGloxdelE13cre Systems (Microbix, St Cloud, MN, USA). Accordingly, a recombinant adenovirus coding shRNA for scramble sequence (Ad-sh-sc) was constructed as a negative control. Cultured cardiomyocytes were infected at a multiplicity of infection (MOI) of 10. After 24 h, cells were treated with palmitate and GLP1 for another 24 h.

### Cell viability assay

Cell viability was measured using a Cell Counting Kit-8 (CCK8) assay kit (Dojindo Bio., Tokyo, Japan). The absorbance was measured at 450 nm using an automated microplate reader.

### Oil red O staining

To measure intracellular lipid accumulation, primary cardiomyocytes were stained by Oil red O dye (Sigma–Aldrich). After fixation using 4% paraformaldehyde, cells were washed and incubated with the Oil red O working solution (3 mg/ml) for 30 min at room temperature. Cells were then counterstained with 2-(4-amidinophenyl)-6-indolecarbamidine dihydrochloride (DAPI) for highlighting nuclei, followed by imaging with an OLYMPUS FV1000 confocal laser-scanning microscope. Oil red O staining was detected under bright field and polarized light.

### TUNEL assay

A TUNEL assay was performed with a Cell Death Detection Kit (Fluorescein; Roche) to detect the apoptosis of isolated cardiomyocytes. TUNEL-positive cells were counted in ten different microscopic fields of at least three independent experiments. The index of apoptosis was presented as the percentage of TUNEL-positive cells to total (DAPI-positive) cells using Image-Pro Plus Software (Rockville, MD, USA).

### Indirect immunofluorescence

Neonatal rat cardiomyocytes cultured on glass coverslips were fixed with 4% paraformaldehyde and permeabilized in 0.1% Triton X-100. After blocking unspecific binding by incubating for 30 min with 1% BSA, cells were incubated overnight at 4 °C in the presence of a rabbit anti-active b-catenin (non-phosphorylated at Ser33/37/Thr41) antibody (1:100; Cell Signalling Technology, Boston, MA, USA) or a rabbit anti-CD36 antibody (1:100; Abcam, Boston, MA, USA). Cells were then incubated at 37 °C for 1 h with an Alexa-Fluor-546 or -488 labelled goat anti-rabbit (1:100; Invitrogen, Life Technologies) secondary antibody and then counterstained with DAPI. Images were obtained with an Olympus FV1000 confocal microscope.

### Subcellular fractionation

For detection of protein expression of b-catenin in the cytosol and nucleus, cardiomyocytes at a density of 1×10^7^ cells/dish were fractionated using the FractionPREP Cell Fractionation Kit (BioVision, Mountain View, CA, USA). To detect the subcellular distribution of CD36, plasma membranes were extracted from 1×10^7^ of cardiomyocytes using a sucrose cushion (38% wt/vol) as previously described (Chabowski *et al*. 2004), and cytosolic proteins were isolated using the Membrane and Cytosol Protein Extraction Kit (Beyotime, Shanghai, China).

### Western blot analysis

Forty micrograms of cellular proteins resolved by SDS–PAGE were immunoblotted as previously described (Ying *et al*. 2012). The primary antibodies (Cell Signalling Technology) were: anti-cleaved caspase-3 (1:1000), anti-cleaved poly (ADP-ribose) polymerase (PARP, 1:1000), anti-non-phosphorylated b-catenin (Ser33/37/Thr41, 1:1000), anti-phosphorylated b-catenin (Ser33/37/Thr41, 1:1000), anti-phosphorylated Akt (Ser473, 1:1000), anti-phosphorylated GSK3b (Ser9, 1:1000), anti-BCL2 (1:1000) and anti-GAPDH (1:1000). Anti-survivin (1:500) and anti-phosphorylated b-catenin (Ser552, 1:500) were from ABclonal (Boston, MA, USA). Anti-CD36 (1:1000) was from Abcam. HRP-conjugated secondary antibodies (1:2000) were from Cell Signalling Technology. Immunoreactive bands were revealed by ECL and visualized by the KODAK Image Station 4000MM PRO. Band intensities were quantified by scanning densitometry (Gel-Doc2000, Bio-Rad) and analyzed with Quantity On (Bio-Rad).

### Statistical analysis

All data were expressed as mean±s.e.m. Statistical significance was analyzed with an unpaired Student's *t*-test. Data were considered significant when *P*<0.05.

## Results

### Effects of palmitate treatment on primary neonatal rat cardiomyocytes

**Figure 1:**
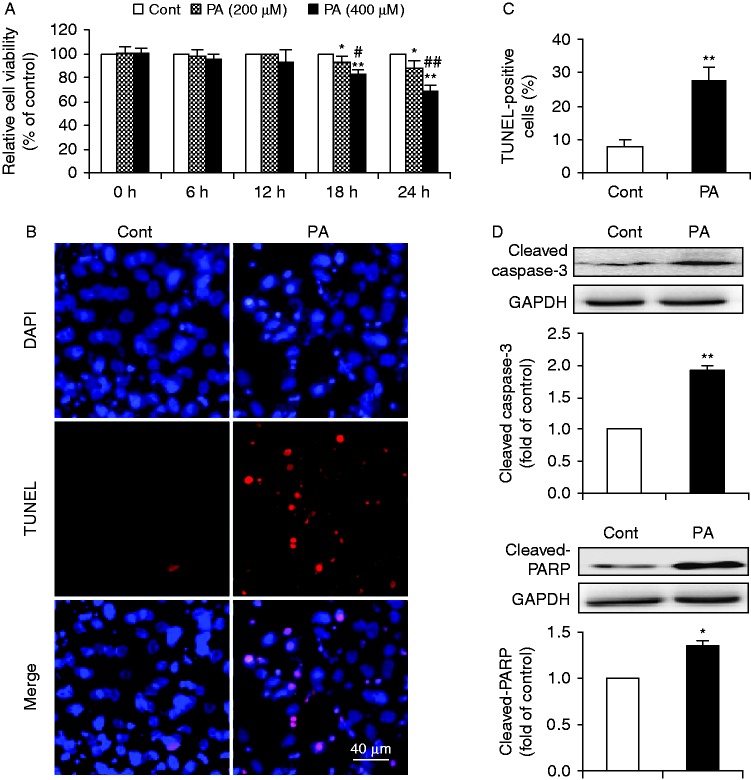
Effects of PA on primary neonatal rat cardiomyocytes. (A) A dose- and time-dependent effect of PA on cardiomyocyte viability. Primary cardiomyocytes were exposed to media containing BSA alone (Cont) or different concentrations (200 and 400 μM) of BSA-conjugated PA for 0, 6, 12, 18, and 24 h respectively. Cell viability was determined by a CCK8 assay. Values are expressed as percentages of cell viability at Cont. Data are means±s.e.m. of three independent experiments in duplicate. *P<0.05 and **P<0.01 vs Cont; ^#^*P*<0.05 and ^##^*P*<0.01 vs 200 μM of PA. PA-induced apoptosis (B, C and D) was further examined by exposure of cardiomyocytes to Cont or PA (400 μM) for 24 h. (B) Representative images of immunostaining for apoptotic (TUNEL-positive, red) cardiomyocytes. Nuclei were labeled with DAPI (blue). Scale bar represents 40 μm. (C) Quantification of apoptotic nuclei by Image-Pro Plus Software. Values are expressed as the percentage of TUNEL-positive cells to total (DAPI-positive) cells. (D) Western blot analysis for cleaved caspase-3 and cleaved-PARP. Intensities of protein expression were quantified, normalized against the level of GAPDH and expressed as fold of protein abundance at control. Data are means±s.e.m. of three independent experiments. *P<0.05 and **P<0.01 vs Cont. Cont, control; PA, palmitate. A full colour version of this figure is available at http://dx.doi.org/10.1530/JME-15-0155.

To determine the toxic effect of palmitate on cardiomyocytes, neonatal rat cardiomyocytes were treated with increasing amounts of BSA-conjugated palmitate (0–400 μM) for 0–24 h. There was a dose- and time-dependent effect of palmitate on cardiomyocyte viability as determined by a CCK8 assay (Fig. 1A). Stimulation of cardiomyocytes with palmitate at 400 μM for 24 h demonstrated a significant increase of toxic effect on cardiomyocytes as compared with control, which was adopted in subsequent experiments. TUNEL assay revealed that cells treated with palmitate showed a significant increase in TUNEL-positive cells, as compared with control (Fig. 1B and C). Moreover, palmitate significantly stimulated caspase-3 activation and proteolytic cleavage of PARP in cardiomyocytes (Fig. 1D). These results demonstrated that palmitate induced apoptosis in primary cardiomyocytes.

### Palmitate attenuated b-catenin signalling in cardiomyocytes

**Figure 2:**
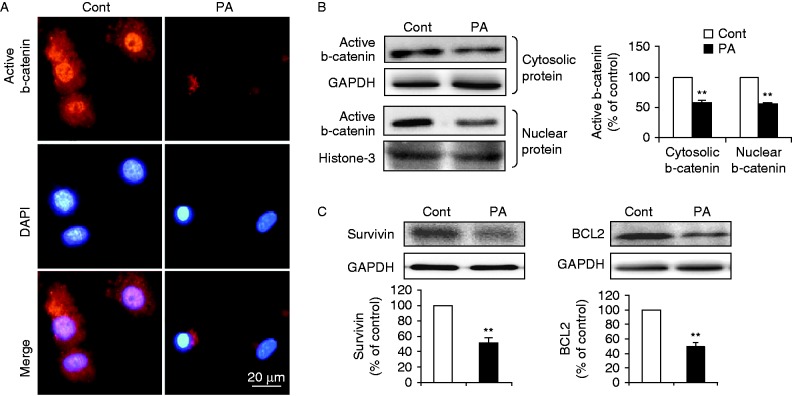
PA attenuated b-catenin signaling in cardiomyocytes. (A) Subcellular localization of active b-catenin in cardiomyocytes incubated with BSA (Cont) or PA (400 μM) was examined by indirect immunofluorescence (red, b-catenin and blue, DAPI; scale bar, 20 μm). (B) Levels of cytosolic and nuclear b-catenin were determined by western blot in subcellular fractions. GAPDH or histone-3 was used as internal control respectively. (C) Protein expression of survivin and BCL2 was examined by western blot. Intensities were quantified and normalized against the level of GAPDH or histone-3 and expressed as percentage of protein abundance at control (B and C). Data are means±s.e.m. of three independent experiments. **P<0.01 vs Cont. Cont, control; PA, palmitate. A full colour version of this figure is available at http://dx.doi.org/10.1530/JME-15-0155.

b-Catenin is a transcriptional regulator of several genes involved in survival/anti-apoptosis. We thus investigated whether palmitate-induced cardiomyocyte apoptosis involved in inactivation of b-catenin signalling. Fluorescence images clearly showed that cells treated with palmitate resulted in markedly reduced cytosolic expression and almost lack of nuclear staining of active (non-phosphorylated) b-catenin, as compared with control (Fig. 2A). Western blot analysis also revealed that the cytosolic and nuclear levels of active b-catenin were both reduced in cardiomyocytes exposed to palmitate compared with cells exposed to control (Fig. 2B). Furthermore, protein levels of survivin and BCL2, the well-known anti-apoptotic factors (Verdecia *et al*. 2000, Salvesen & Duckett 2002, Kaga *et al*. 2006), were significantly downregulated in palmitate-treated cells compared with control (Fig. 2C). Of note, survivin and BCL2 have been demonstrated as downstream molecules transcriptionally activated by b-catenin in cardiomyocytes (Hahn *et al*. 2006). Therefore, our data indicated that palmitate treatment attenuated b-catenin signalling, which may in turn lead to the induction of apoptosis in cardiomyocytes.

### GLP1 antagonized palmitate-induced apoptotic death of cardiomyocytes

**Figure 3:**
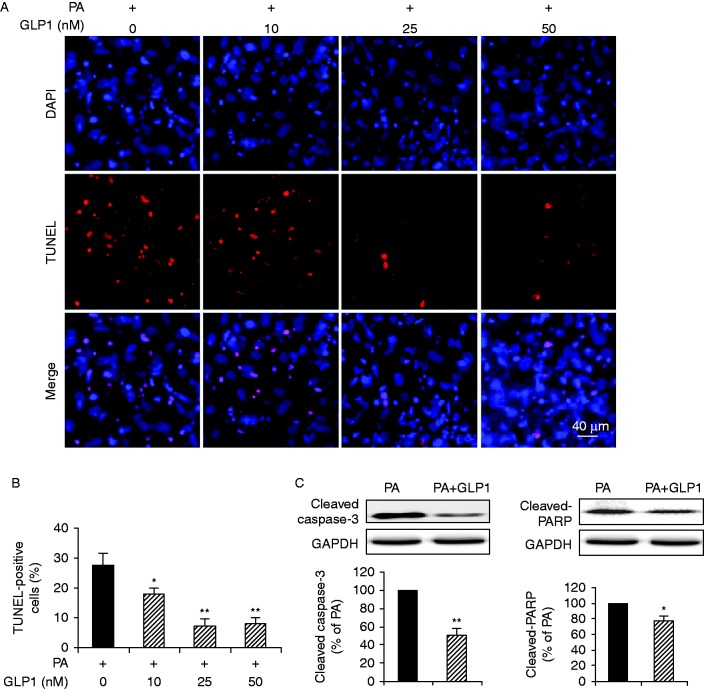
GLP1 antagonized palmitate-induced apoptosis of cardiomyocytes. Isolated cardiomyocytes were incubated with PA for 24 h in the absence or presence of different concentrations of GLP1 (10–50 nM). (A) Representative immunostaining for TUNEL-positive (red) cells. Nuclei were labeled with DAPI (blue). Scale bar represents 40 μm. (B) Apoptotic cells were quantified and expressed as the percentage of TUNEL-positive cells to DAPI-positive cells. (C) Western blot analysis of cleaved caspase-3 and cleaved-PARP in cardiomyocytes without or with GLP1 (25 nM) treatment under PA stress. Intensities were quantified and normalized against the level of GAPDH and expressed as percentage of protein abundance under PA stimulus. Data are means±s.e.m. of three independent experiments. *P<0.05 and **P<0.01 vs PA. PA, palmitate. A full colour version of this figure is available at http://dx.doi.org/10.1530/JME-15-0155.

GLP1 has been described to exhibit anti-apoptotic effects in a few models (Bose *et al*. 2005, Poornima *et al*. 2008, Timmers *et al*. 2009, Ravassa *et al*. 2011, Noyan-Ashraf *et al*. 2013). We first confirmed the protective effect of GLP1 in our experimental set up. Neonatal rat cardiomyocytes were incubated with palmitate for 24 h in the absence or presence of different concentrations of GLP1 (10–50 nM). Palmitate increased TUNEL-positive cells after 24 h incubation, whereas GLP1 dose-dependently suppressed apoptosis in palmitate-treated cardiomyocytes (Fig. 3A and B). Compared with palmitate, cells treated with GLP1 at 25 nM showed a significant decrease in TUNEL-positive cells. The anti-apoptotic effect of GLP1 at 25 nM was comparable to that at 50 nM (Fig. 3B). Thus, 25 nM of GLP1 was adopted for further experiments. As shown in Fig. 3C, GLP1 (25 nM) treatment markedly abrogated palmitate-induced cleavage of caspase-3 and PARP. These data validated that the induction of apoptosis in palmitate-stressed cardiomyocytes were prevented by GLP1 treatment.

### GLP1 restored b-catenin signalling attenuated by palmitate

**Figure 4:**
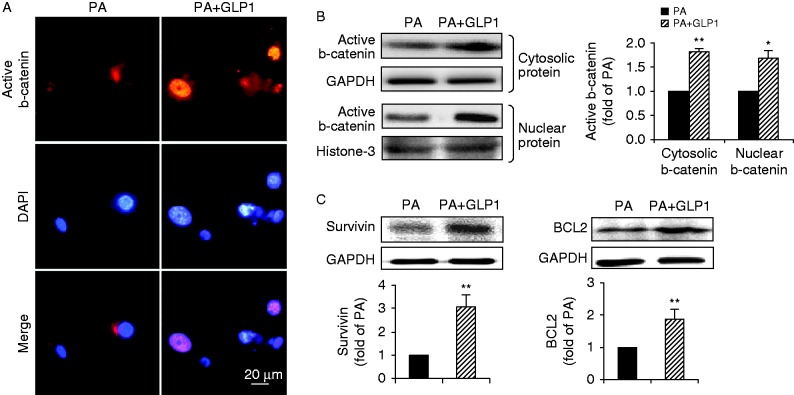
GLP1 restored the attenuated b-catenin signaling induced by PA. (A) Representative images of subcellular distribution of active b-catenin (red, b-catenin and blue, DAPI; scale bar, 20 μm) in cardiomyocytes incubated with PA in the absence or presence of GLP1 (25 nM). (B and C) Western blot assay for cytosolic and nuclear b-catenin, survivin and BCL2. Intensities were quantified and normalized against the level of GAPDH or histone-3 and expressed as fold changes of protein abundance under PA stimulus. Data are means±s.e.m. of three independent experiments. *P<0.05 and **P<0.01 vs PA. PA, palmitate. A full colour version of this figure is available at http://dx.doi.org/10.1530/JME-15-0155.

We next determined whether GLP1 could restore the attenuated b-catenin signalling induced by palmitate. As shown in Fig. 4A, GLP1 prevented the palmitate-mediated loss of nuclear accumulation of active b-catenin in cardiomyocytes. Accordingly, treatment with GLP1 also significantly restored the accumulation of active b-catenin in the cytosol and nucleus as determined by western blot (Fig. 4B). Furthermore, stabilization of b-catenin by GLP1 significantly alleviated the suppressing effect of palmitate on protein expression of target survivin and BCL2 in cardiomyocytes (Fig. 4C). These findings revealed that GLP1 can restore palmitate-mediated attenuation of b-catenin signalling in cardiomyocytes.

### b-Catenin was required for the anti-apoptotic effect of GLP1 in response to palmitate stress

**Figure 5:**
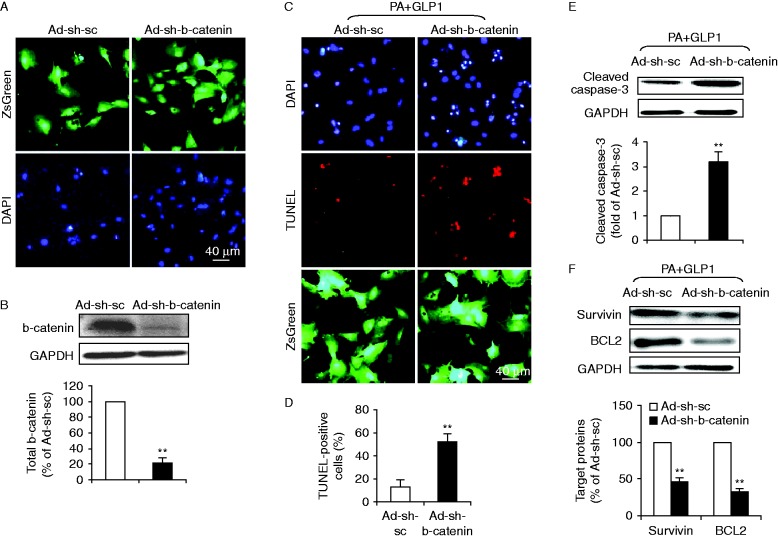
b-Catenin was required for GLP1-mediated anti-apoptotic effects upon lipotoxicity. A recombinant adenovirus coding shRNA for b-catenin (Ad-sh-b-catenin) or for scramble sequences (Ad-sh-sc) was constructed to express ZsGreen protein as a marker for the identification of infected cells. Cultured cardiomyocytes were infected with respective adenoviruses at a MOI of 10. After 24 h, cells were treated with palmitate (PA) and GLP1 (25 nM) for another 24 h. (A) Transduction efficiency of recombinant adenoviruses was assayed by ZsGreen fluorescence analysis (green, ZsGreen and blue, DAPI; scale bar, 40 μm) 1 day after infection. (B) The suppression efficiency of b-catenin using shRNA was determined by western blot analysis for total b-catenin expression. (C, D, E and F) The effect of b-catenin silencing on GLP1 action in PA-treated cardiomyocytes was further assessed. (C) Representative images showed TUNEL staining for apoptotic cells (red, TUNEL and blue, DAPI; green, ZsGreen; scale bar, 40 μm). (D) Quantification of apoptotic nuclei was expressed as the percentage of TUNEL-positive to DAPI-positive cells. (E and F) Western blot analysis for cleaved caspase-3, survivin and BCL2. Intensities of protein expression were quantified, normalized against the level of GAPDH and expressed as relative changes to protein abundance in cardiomyocytes infected with scramble control (Ad-sh-sc). Data are means±s.e.m. of three independent experiments. **P<0.01 vs Ad-sh-sc. A full colour version of this figure is available at http://dx.doi.org/10.1530/JME-15-0155.

To further determine whether b-catenin is required for the anti-apoptotic effect of GLP1 on cardiomyocytes, we adopted a shRNA approach to knockdown b-catenin. Neonatal rat cardiomyocytes were infected with an adenovirus coding for shRNA constructs against b-catenin (Ad-sh-b-catenin) or shRNA for scramble sequences (Ad-sh-sc). The transduction efficiency with recombinant adenoviruses after 24 h was ∼95% as determined by the expression of ZsGreen in cardiomyocytes infected by Ad-sh-b-catenin or Ad-sh-sc. Infection with the adenovirus had no detectable effect on cardiomyocyte morphology and viability (Fig. 5A). Western blot analysis showed that total b-catenin levels were reduced by 78.1±6.6% (*P*<0.01) in Ad-sh-b-catenin-infected cardiomyocytes, compared with scramble control Ad-sh-sc (Fig. 5B). We next assessed the effect of b-catenin silencing on GLP1 action in palmitate-treated cardiomyocytes. Our results showed that in response to GLP1, knockdown of b-catenin significantly increased the number of TUNEL-positive cells (Fig. 5C and D) upon lipotoxicity, compared to Ad-sh-sc-infected groups. In consistent with TUNEL-staining, b-catenin downregulation also abrogated the inhibiting effect of GLP1 on palmitate-promoted caspase-3 activation (Fig. 5E). Moreover, silencing of b-catenin abolished GLP1-restored expression of b-catenin target proteins, survivin and BCL2 (Fig. 5F). Together, these data validated that b-catenin is a requisite for GLP1-mediated anti-apoptotic effects upon lipotoxicity.

### The anti-apoptotic effect of GLP1 occurred via GLP1R

**Figure 6:**
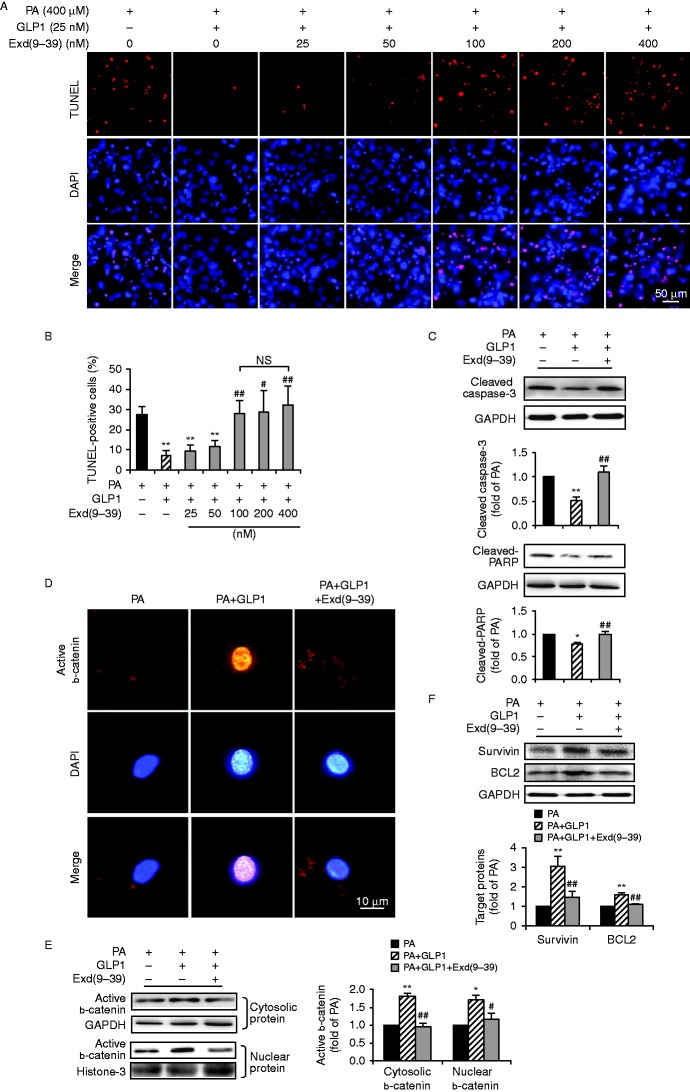
The anti-apoptotic effect of GLP1 occurred via the GLP1 receptor. (A and B) Isolated cardiomyocytes cultured at PA (400 μM) for 24 h were treated with GLP1 (25 nM) alone, or in combination with an increasing amount (25–400 nM) of Exd(9–39). Representative diagrams showed TUNEL staining of apoptotic cells (red, TUNEL and blue, DAPI; scale bar, 50 μm) (A). Quantification of apoptotic nuclei were expressed as the percentage of TUNEL-positive cells to DAPI-positive cells (B). 25 nM of GLP1 and 100 nM of Exd(9–39) were adopted in subsequent experiments (C, D, E and F). Western blot analysis for cleaved caspase-3 and cleaved-PARP (C); cytosolic and nuclear b-catenin (E); survivin and BCL2 (F). Intensities were quantified and normalized against the level of GAPDH or histone-3 and expressed as fold changes of protein abundance under PA stimulus. Data are means±s.e.m. of three independent experiments. *P<0.05 and **P<0.01 vs PA; ^#^*P*<0.05 and ^##^*P*<0.01 vs PA+GLP1. Representative images of subcellular localization of active b-catenin (red, b-catenin and blue, DAPI; scale bar, 10 μm) (D) in cardiomyocytes. PA, palmitate; Exd(9–39), exendin-(9–39); NS, no significant difference. A full colour version of this figure is available at http://dx.doi.org/10.1530/JME-15-0155.

GLP1 acts through its cognate G-protein-coupled receptor, GLP1R, which is expressed in several tissues including the heart (Bullock *et al*. 1996, Ahrén 2004). To determine whether GLP1 protected cardiomyocytes against palmitate-induced apoptosis via GLP1R, cardiomyocytes cultured at palmitate for 24 h were treated with GLP1 (25 nM) alone, or in combination with an increasing amount (25–400 nM) of exendin-(9–39), a specific and competitive antagonist of GLP1R. TUNEL staining showed that co-administration of GLP1 with 100 nM of exendin-(9–39) led to an increase of apoptotic nuclei comparable to that with 200 or 400 nM of exendin-(9–39) (Fig. 6A and B). Thus, combination of GLP1 at 25 nM and exendin-(9–39) at 100 nM was chosen in further experiments. Our results further validated that application of exendin-(9–39) markedly abrogated the anti-apoptotic effect of GLP1 on cardiomyocytes under palmitate stimulus, leading to increased activation of caspase-3 and PARP (Fig. 6C). Moreover, inhibition of GLP1R abolished the GLP1-restored cellular accumulation and nuclear translocation of active b-catenin (Fig. 6D and E), and protein expression of survivin and BCL2 (Fig. 6F). Collectively, these data demonstrated that the anti-apoptotic effect of GLP1 through activation of b-catenin signalling occurred in a GLP1R-dependent manner.

### Rescue of b-catenin signalling by GLP1 in palmitate-treated cardiomyocytes was mediated by Akt/GSK3b pathway in a GLP1R-dependent manner

**Figure 7:**
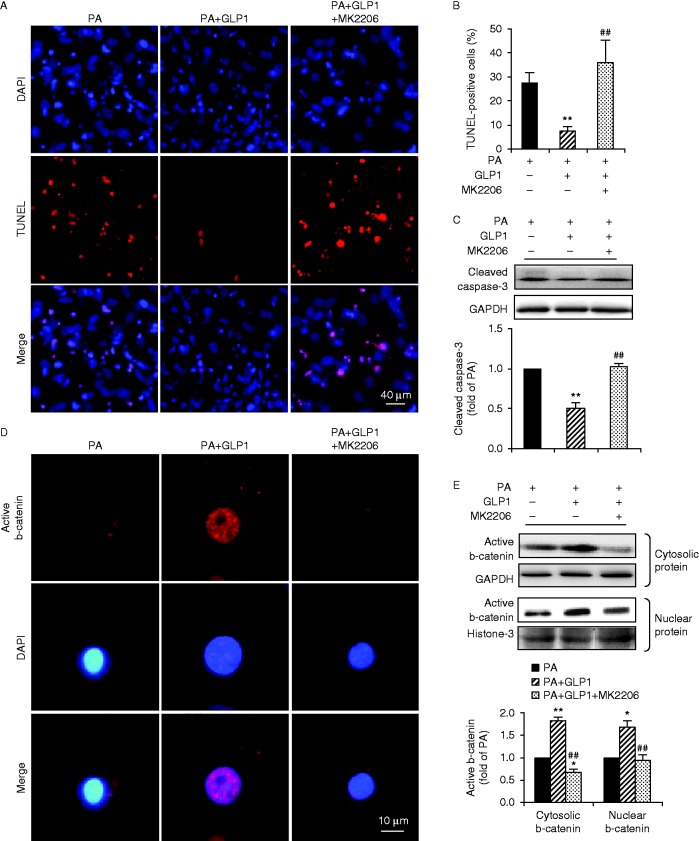
GLP1-mediated anti-apoptotic effects were abolished when Akt was inhibited. Cultured cardiomyocytes were incubated with PA (400 μM) for 24 h in the absence or presence of GLP1 (25 nM) alone, or in combination of GLP1 (25 nM) with an Akt inhibitor MK2206 (50 nM). (A) Apoptotic cardiomyocytes were examined by TUNEL staining (red, TUNEL and blue, DAPI; scale bar, 50 μm). (B) Numbers of apoptotic cells were quantified and expressed as the percentage of TUNEL-positive to DAPI-positive cells. (C) Levels of cleaved caspase-3 were analyzed by western blot and quantified by densitometry. Distribution of active b-catenin in cardiomyocytes was determined by immunostainning (red, b-catenin and blue, DAPI; scale bar, 10 μm) (D) and levels of cytosolic and nuclear b-catenin were further examined by western blot (E). Intensities were quantified and normalized against the level of GAPDH or histone-3 and expressed as fold changes of protein abundance under PA stimulus. Data are means±s.e.m. of three independent experiments. *P<0.05 and **P<0.01 vs PA; ^##^*P*<0.01 vs PA+GLP1. PA, palmitate. A full colour version of this figure is available at http://dx.doi.org/10.1530/JME-15-0155.

**Figure 8:**
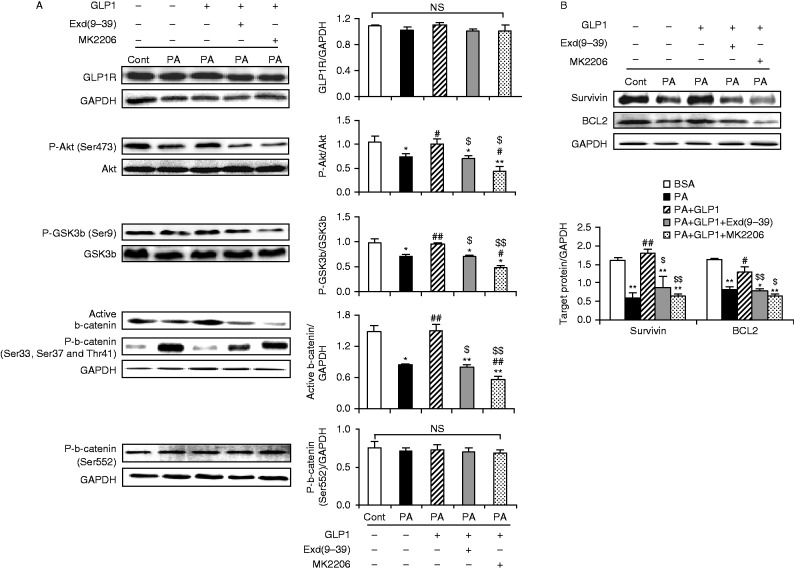
Reversal of b-catenin signaling by GLP1 was mediated via GLP1R/Akt/GSK3b/ pathways in PA-stressed cardiomyocytes. Cardiomyocytes were incubated with PA (400 μM) for 24 h in the absence or presence of GLP1 (25 nM) alone, or in combination of GLP1 with Exd(9–39) (100 nM) or MK2206 (50 nM). (A and B) Western blot analysis for GLP1R, phosphorylated-Akt (Ser473), total Akt, phosphorylated-GSK3b (Ser9), total GSK3b, active b-catenin, phosphorylated-b-catenin (Ser33/37/Thr41), phosphorylated-b-catenin (Ser552), survivin, BCL2 and GAPDH. Intensities were quantified and normalized against the level of total proteins (Akt and GSK3b) or GAPDH. Data are means±s.e.m. of three independent experiments. *P<0.05 and **P<0.01 vs BSA control; ^#^*P*<0.05 and ^##^*P*<0.01 vs PA; ^$^*P*<0.05 and ^$$^*P*<0.01 vs PA+GLP1. PA, palmitate; Exd(9–39), exendin-(9–39); NS, no significant difference.

Akt activation leads to GSK3b phosphorylation (Ser9) and inactivation and thus the stabilization of b-catenin. We first investigate whether Akt is required for the beneficial effect of GLP1 on palmitate-treated cardiomyocytes. Cardiomyocytes were incubated with palmitate for 24 h in the absence or presence of GLP1 alone, or in combination of GLP1 with MK2206 (an Akt inhibitor, 50 nM). Compared to GLP1 alone, co-administration of MK2206 failed to suppress the palmitate-induced apoptosis of cardiomyocytes, which led to a significant increase in TUNEL-positive cells (Fig. 7A and B) and caspase-3 cleavage (Fig. 7C). Moreover, inhibition of Akt by MK2206 completely abrogated GLP1-restored intracellular accumulation and nuclear translocation of active b-catenin (Fig. 7D and E). The effect of GLP1 on restoring protein expression of survivin and BCL2 was also significantly alleviated when Akt was inhibited (Fig. 8B). Collectively, these observations revealed that Akt was required for GLP1-mediated activation of nuclear b-catenin signalling in palmitate-treated cardiomyocytes.

The modulation of upstream signals of b-catenin in the cardiomyocytes was further investigated. Compared with the BSA control, palmitate significantly reduced the phosphorylation of Akt (Ser473) and increased GSK3B activation as demonstrated by the impairment of phosphorylated-Ser9–GSK3B expression, which led to a dramatically decreased stability of b-catenin as demonstrated by reduced expression of active b-catenin (non-phosphorylated at Ser33/37/Thr41) (Fig. 8A). As a result, the downstream targets of nuclear b-catenin, survivin and BCL2, were distinctly downregulated by palmitate (Fig. 8B). In contrast, GLP1 treatment significantly increased the Akt phosphorylation and inhibited GSK3B activation, leading to a remarkably improved accumulation of b-catenin and increased expression of b-catenin target protein as compared to palmitate-treated group. However, these stimulatory effects of GLP1 on cardiomyocytes were largely blocked by an Akt inhibitor, MK2206, or a GLP1R antagonist, exendin-(9–39) (Fig. 8A and B). Interestingly, the protein level of GLP1R remained unchanged at the respective condition (Fig. 8A). Taken together, our data demonstrated that GLP1 could protect cardiomyocytes from palmitate-induced apoptosis via activation of Akt/GSK3B/b-catenin in a GLP1R-dependent manner.

### GLP1 prevented CD36-mediated intracellular lipid accumulation in palmitate-stressed cardiomyocytes via the GLP1R/Akt axis

**Figure 9:**
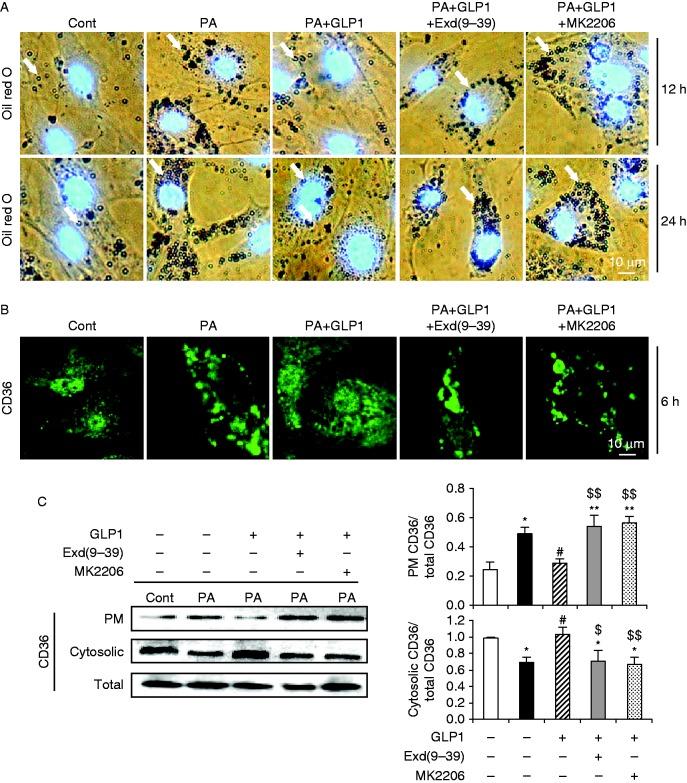
GLP1 prevented CD36-mediated intracellular lipid accumulation in PA-stressed cardiomyocytes via the GLP1R/Akt axis. Cardiomyocytes were incubated with PA (400 μM) in the absence or presence of GLP1 (25 nM) alone, or in combination of GLP1 with Exd(9–39) (100 nM) or MK2206 (50 nM). (A) Oil red O staining for intracellular neutral lipids (red–brown, indicated by white arrow) accumulated in cardiomyocytes after the respective 12 h (top) or 24 h (bottom) incubation. Nuclei were counterstained by DAPI (blue). Scale bar represents 10 μm. (B) Subcellular distribution of CD36 (green, CD36; scale bar, 10 μm) in cardiomyocytes after 6 h incubation was examined by indirect immunofluorescence. (C) Levels of plasma membrane, cytosolic and total CD36 were determined by western blot. Intensities were quantified and normalized against the level of total CD36. Data are means±s.e.m. of three independent experiments. *P<0.05 and **P<0.01 vs BSA control; ^#^*P*<0.05 vs PA; ^$^*P*<0.05 and ^$$^*P*<0.01 vs PA+GLP1. PA, palmitate; Exd(9–39), exendin-(9–39); PM, plasma membrane. A full colour version of this figure is available at http://dx.doi.org/10.1530/JME-15-0155.

Last, we also assessed the effect of palmitate on intracellular lipid accumulation in cultured cardiomyocytes, as intramyocardial accumulation of triacylglycerol metabolites may contribute to diabetic/lipotoxic cardiomyopathy (Fang *et al*. 2004). Oil red O staining revealed that exposure of cardiomyocytes to palmitate for either 12 or 24 h caused a robust increase in neutral lipid accumulation, as compared to control (Fig. 9A). As reported, uptake of free fatty acid is mainly mediated by the fatty acid transporter CD36 through translocation of CD36 from an intracellular pool to the plasma membrane in cardiomyocytes (Luiken *et al*. 2002, Koonen *et al*. 2005). We then examined the subcellular distribution of CD36 and found that incubation with palmitate for as early as 6 h induced some of the CD36 to the plasma membrane while concomitantly lowering the intracellular depot of CD36, as compared to control (Fig. 9B). The augmented membrane presence and reduced cytosolic deposition of CD36 after palmitate treatment for 6 h was confirmed by western blot (Fig. 9C). In contrast, GLP1 treatment suppressed palmitate-induced intracellular lipid accumulation (Fig. 9A), accompanied by a markedly reduced membrane translocation of CD36 in cardiomyocytes (Fig. 9B and C). However, these beneficial effects of GLP1 were mostly blunted by MK2206 or exendin-(9–39) (Fig. 9A, B and C). Notably, no significant change in total protein content of CD36 was observed at the respective condition (Fig. 9C). Thus, our data suggested that GLP1 could prevent palmitate-induced CD36 membrane translocation and lipid accumulation in cardiomyocytes in a GLP1R/Akt-dependent manner.

## Discussion

Our present findings first highlight the importance of GLP1-mediated b-catenin signalling in protecting cardiomyocytes from palmitate-induced apoptosis. To our knowledge, this study represents the first demonstration that b-catenin signalling was attenuated in a model of palmitate-induced cardiomyocyte apoptosis, which was restored by GLP1 treatment.

Although previous studies have described the association of stabilized b-catenin and cell survival in several models *in vitro* (Bergmann *et al*. 2004, Venkatesan *et al*. 2010) and *in vivo* (Hahn *et al*. 2006, Kaga *et al*. 2006, Thirunavukkarasu *et al*. 2008), little is known regarding the role of b-catenin in the context of diabetic/lipotoxic cardiomyopathy. Our results from the present study showed that palmitate induced decreased accumulation of b-catenin in the cytosol and nucleus of isolated cardiomyocytes, which was accompanied by the downregulated expression of survivin and BCL2, the important downstream target genes of b-catenin (Behrens *et al*. 1996, Miller *et al*. 1999). Recent studies have also suggested that survivin and BCL2 are transcriptionally activated by b-catenin in cardiomyocytes (Hahn *et al*. 2006). Of note, survivin is a member of the apoptosis inhibiting gene family (Verdecia *et al*. 2000, Salvesen & Duckett 2002) and exerts its pro-survival effects via physically associating with the active form of executioner caspase such as caspase-3 *in vitro* and prevents a cascade of caspase cleavage and activation amplification (Tamm *et al*. 1998). The expression of survivin has been reported to be inversely associated with the presence of dilated cardiomyopathy and cardiomyocyte apoptosis (Santini *et al*. 2004). BCL2 is one of the representative molecules to inhibit mitochondria-dependent apoptotic pathway, which inhibits the release of cytochrome c and Smac/DIABLO homolog from mitochondria into cytosol (Zamzami *et al*. 1998, Fesik & Shi 2001). Thus, the downregulation of survivin and BCL2 by palmitate observed in this study may have a direct effect on mitochondrial membrane pore formation and consequent activation of caspase-3 and PARP activity, therefore leading to activation of the intrinsic apoptotic pathway. Therefore, it is conceivable that the palmitate-attenuated b-catenin signalling described in the present study might be relevant to the increased apoptosis of cardiomyocytes. This notion is partially supported by some other studies in ischemic myocardium models, in which depletion of cytosolic b-catenin resulted in increased apoptosis and infarct size (Thirunavukkarasu *et al*. 2008), whereas forced expression of b-catenin led to reduced apoptotic cardiomyocytes and infarct size (Hahn *et al*. 2006). In addition, the observation of decreased b-catenin levels in failing human hearts (Schumann *et al*. 2000) also supports our result.

GLP1 has already been described as an anti-apoptotic factor in a few cardiac disease models *in vitro* (Ravassa *et al*. 2011, Noyan-Ashraf *et al*. 2013) and *in vivo* (Bose *et al*. 2005, Timmers *et al*. 2009). In this study, we confirmed that GLP1 protected isolated rat cardiomyocytes from palmitate-induced apoptosis as previously reported (Noyan-Ashraf *et al*. 2013). We also found that GLP1 can antagonize the palmitate-mediated suppression of b-catenin nuclear translocation and protein expression of survivin and BCL2, suggesting that GLP1 can restore the b-catenin signalling attenuated by palmitate. Importantly, our results further showed that silencing of b-catenin by specific shRNA abolished the anti-apoptotic effect of GLP1 and reduced expression of anti-apoptotic molecules such as survivin and BCL2. Therefore, our findings provide solid evidences that b-catenin signalling provides a requisite link in the anti-apoptotic effect of GLP1 under palmitate stress.

It is noteworthy that the upstream molecules and mechanisms responsible for GLP1-associated cardiac protection remain elusive in diabetes and obesity. A recent report revealed that inactivation of GSK3B may be involved in GLP1-offered protective effects in high-fat diet mice (Noyan-Ashraf *et al*. 2013). Of potential interest, GSK3B was an essential regulator of b-catenin, which constitutively phosphorylates cytosolic b-catenin (Ser33/37/Thr41) for proteasomal degradation (Huelsken & Birchmeier 2001). Inactivation of GSK3B through phosphorylation (Ser9) by survival (PI3K/Akt and Wnt) pathway leads to b-catenin stabilization and nuclear translocation (Almeida *et al*. 2005). In this study, we have shown that GLP1-mediated prevention of palmitate-induced apoptosis of cardiomyocytes and attenuation of b-catenin signalling was accompanied by the increased phosphorylation of Akt (Ser473) and GSK3B (Ser9, inactivated form), and this effect of GLP1 was abrogated when blocking the Akt pathway with the Akt-specific inhibitor MK2206. Thus, another novel finding of our present study is that GLP1 requires activation of the Akt/GSK3B/b-catenin pathway to prevent palmitate-induced apoptosis in cardiomyocytes.

Data from a previous study also implicates Akt-dependent b-catenin signalling pathways as the mechanism by which the GLP1 analogue exerts its action on enhancing proliferation of pancreatic b cells (Liu & Habener 2008), where, however, GLP1-mediated activation of Akt/b-catenin was independent of GSK3B. The activation of b-catenin without GSK3B inhibition was supported by other reports that phosphorylation of b-catenin at Ser552 through Akt or cAMP-dependent protein kinase promotes the transcriptional activity of b-catenin without alteration of b-catenin degradation (Taurin *et al*. 2006). Nevertheless, no significant difference in protein levels of b-catenin (phosphorylated at Ser552) was found in control and palmitate-stressed cardiomyocytes without or with GLP1 treatment (Fig. 8A). It would seem, therefore, that in the context of cardiomyocytes, GSK3b inactivation turns out to be a critical effector mediating the anti-apoptotic effect of b-catenin signalling as reported in the present and previous studies (Haq *et al*. 2003, Bergmann *et al*. 2004, Kaga *et al*. 2006, Wu *et al*. 2012). However, these observations are apparently in disagreement with the latest published report (Xi *et al*. 2015), where the activation of GSK3B/b-catenin was associated with progressive cardiomyocyte apoptosis in diabetic rats. The difference in disease model used may explain these controversial results. Xi *et al*. adopted a single injection of high-dose streptozotocin to induce a rat model of type 1 diabetes usually associated with the absence of hyperlipidemia. Recent studies have shown that ceramide, which can be derived from palmitate by *de novo* synthesis (Merrill & Jones 1990), can lead to Akt inhibition (Summers *et al*. 1998, Zhou *et al*. 1998). Our data also validated that palmitate reduced the phosphorylation of Akt (Ser473, Fig. 8A). Thus, it is tempting to ascribe the Akt inhibition by palmitate to the attenuated GSK3B/b-catenin signalling and increased cardiomyocyte apoptosis in our model.

Interestingly, our data also revealed that palmitate can augment CD36 membrane translocation and intracellular lipid accumulation, which preceded the onset of cell death, as no significant decrease in cell viability was detected up to 12 h (Fig. 1A). Previous studies have shown that targeting CD36 to the plasma membrane by palmitate was largely a consequence of increased nuclear entry of FoxO1, which can regulate fatty acid uptake and triglyceride storage (Puthanveetil *et al*. 2011), while the nuclear exclusion of FoxO1 could be induced by Akt activation (Glauser & Schlegel 2007). Thus, it seems plausible that palmitate, first taken up into cells by passive diffusion, inhibits Akt activation via *de novo* synthesis of ceramide, thus suppresses FoxO1 nuclear exclusion and induces CD36 membrane translocation, leading to increased palmitate uptake and lipid accumulation. By contrast, GLP1 can prevent palmitate-induced CD36 membrane translocation and lipid accumulation in cardiomyocytes via activation of Akt, since inhibition of Akt by MK2206 blocked these beneficial effects of GLP1 in response to palmitate.

Also, data from our study show that GLP1-mediated cardioprotection and activation of Akt/GSK3b/b-catenin signalling was abolished by using the GLP1R antagonist exendin-(9–39). The GLP1R-dependent protective effect of GLP1 observed in our study was consistent with some previous studies (Liu & Habener 2008, Noyan-Ashraf *et al*. 2013) but contradictory with others (Ban *et al*. 2008, Ravassa *et al*. 2011). It is currently unknown what causes this discrepancy.

**Figure 10:**
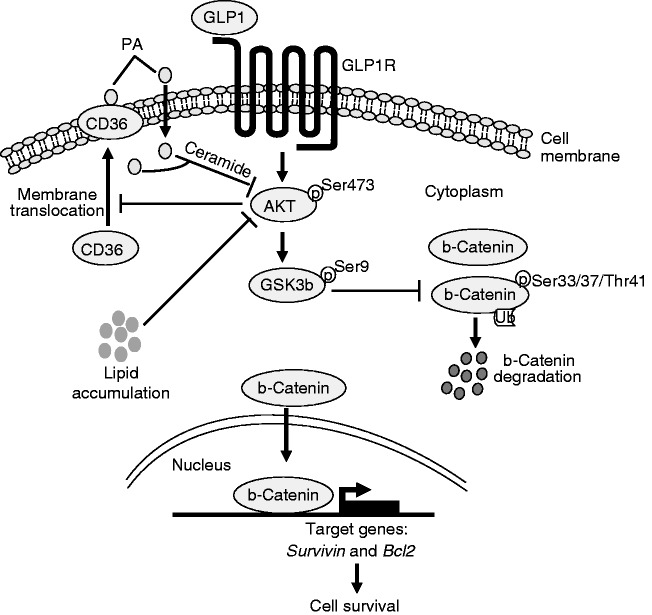
Schematic of a proposed model for GLP1-mediated signaling pathway protecting cardiomyocytes from PA-induced lipotoxicity. PA, palmitate.

Based on the present findings and those published before (Kaga *et al*. 2006, Liu & Habener 2008), we outlined a hypothetical model that explains our findings (Fig. 10). Thus, Akt inhibition by exposure of cardiomyocytes to palmitate would promote membrane translocation of CD36, and thus augment membrane CD36-mediated palmitate uptake and lipid accumulation on the early signalling events. The increased uptake of palmitate would further exacerbate Akt inactivation. Moreover, inhibition of Akt would lead to GSK3B hyper-activation, and subsequent b-catenin degradation, which at least partly account for the increased cell apoptosis. Importantly, GLP1 could activate Akt via GLP1R, leading to prevention of membrane CD36-mediated palmitate uptake and lipid accumulation, as well as GSK3b inhibition and b-catenin accumulation in the cytosol and nucleus, which triggers the expression of required genes, such as survivin and BCL2, for cell survival. This suggestion awaits further confirmation in more complex physiological disease processes *in vivo*.

In summary, our present study demonstrated for the first time that i) the attenuated b-catenin signalling may contribute to palmitate-induced cardiomyocyte apoptosis; ii) a restoration of b-catenin signalling by GLP1 is sufficient to protect cardiomyocytes from palmitate-induced lipotoxicity; iii) the anti-apoptotic effects of GLP1 are dependent on downstream activation of b-catenin signalling and mediated by a GLP1R/Akt/GSK3b axis; iv) GLP1 can prevented palmitate-induced CD36 membrane translocation and intracellular lipid accumulation in cardiomyocytes via activation of Akt. Our findings suggest a potential therapeutic target for b-catenin signalling and also a prospective beneficial role for GLP1 in palmitate-induced cardiomyocyte apoptosis. These results may also provide implications in the treatment of diabetic/lipotoxic cardiomyopathy.

## Author contribution statement

Y Y conceived and designed the study, carried out the studies, performed data analyses and interpretation, and wrote the manuscript. H Z participated in study design, carried out the experiments, performed data collection, and analyses. Z L participated in research concept and critical reading of manuscript. X M provided critical comments on this study. S L participated in the *in vitro* experiments.

## Figures and Tables

**Table 1:** shRNA sequences used for construction of recombinant adenoviruses

**Target**	**Sequences** (5′–3′)
Sh-b-catenin-1	F: GATCCGCAGCAATCTTACCTGGATTTCAAGAGAATCCAGGTAAGATTGCTGCTTTTTT
	R: AATTAAAAAAGCAGCAATCTTACCTGGATTCTCTTGAAATCCAGGTAAGATTGCTGCG
Sh-b-catenin-2	F: GATCCGCCACTAATGTCCAGCGCTTTTCAAGAGAAAGCGCTGGACATTAGTGGTTTTTT
	R: AATTAAAAAACCACTAATGTCCAGCGCTTTCTCTTGAAAAGCGCTGGACATTAGTGGCG
Sh-b-catenin-3	F: GATCCGCCACAGGACTACAAGAAATTCAAGAGATTTCTTGTAGTCCTGTGGCTTTTTT
	R: AATTAAAAAAGCCACAGGACTACAAGAAATCTCTTGAATTTCTTGTAGTCCTGTGGCG
Sh-scramble	F: CCGGTGCTTCGACATTTAACCAATTTCAAGAGAATTGGTTAAATGTCGAAGCTTTTTTG
	R: AATTCAAAAAAGCTTCGACATTTAACCAATTCTCTTGAAATTGGTTAAATGTCGAAGCA

**Table 2:** Primer sequences used for quantitative real-time PCR

**Gene**	**Sequences** (5′–3′)
*b-catenin*	F: TCCCAGTCCTTCACGCAAGAG
	R: GTGGCAAGTTCCGCGTCATC
